# Questionnaire survey on cardiologists’ view and management of coronary microvascular disease in clinical practice

**DOI:** 10.1007/s12471-019-1274-x

**Published:** 2019-04-12

**Authors:** E. Aribas, S. E. Elias-Smale, D. J. Duncker, J. J. Piek, M. A. Ikram, Y. Appelman, J. E. Roeters van Lennep, M. Kavousi

**Affiliations:** 1 Department of Epidemiology, Erasmus MC, University Medical Centre Rotterdam, Rotterdam, The Netherlands; 2 Department of Cardiology, Radboud University Medical Centre, Nijmegen, The Netherlands; 3 Department of Cardiology, Erasmus MC, University Medical Centre Rotterdam, Rotterdam, The Netherlands; 4 Department of Cardiology, Amsterdam University Medical Centres, location AMC, Amsterdam, The Netherlands; 5 Department of Cardiology, Amsterdam University Medical Centres, location VU University Medical Centre, Amsterdam, The Netherlands; 6 Department of Internal Medicine, Erasmus MC, University Medical Centre, Rotterdam, The Netherlands

**Keywords:** Coronary microvascular diseas, Survey questionnaire, Opinion poll, Practice guidelines

## Abstract

**Objective:**

We aimed to assess the opinion of Dutch cardiologists on coronary microvascular disease (CMD) and its management in clinical practice, and to assess the need for a CMD guideline among Dutch cardiologists.

**Methods:**

We developed an online questionnaire including different aspects of CMD which was reviewed by an expert panel. The questionnaire was distributed by e‑mail among all members of the Dutch Society of Cardiology.

**Results:**

A total of 103 cardiologists (70% male) completed the questionnaire (response rate: 10%). Median age and years of experience as a cardiologist were 49 ± 15 and 12 ± 12 years, respectively. Overall, 93% of the cardiologists had considered the CMD diagnosis, 85% had ever made such a diagnosis, 90% had treated a patient with CMD, and 61% had referred patients to tertiary care. The median (interquartile range) self-rated knowledge level was 7.0 (2.0) (scale of 0–10). 84% rated their knowledge as sufficient (>5.5) and 58% viewed CMD as a disease entity. Overall, 61% and 17%, respectively, agreed that evidence-based diagnostic and treatment modalities for CMD do not exist, while 56% believed that CMD patients have a higher risk for cardiovascular disease and mortality. Finally, 82% of the responders stated that a CMD guideline is needed, and 91% wanted to receive the guideline once developed.

**Discussion:**

Fifty-eight per cent of the responders recognise CMD as a separate disease entity. Our study underscores the need for a dedicated CMD guideline for Dutch cardiology practice. However, the response rate was low (10%), and it is likely that mainly cardiologists interested in CMD have participated in our study.

**Electronic supplementary material:**

The online version of this article (10.1007/s12471-019-1274-x) contains supplementary material, which is available to authorized users.

## What’s new?

- This is the first study investigating the opinion of cardiologists in the Netherlands on coronary microvascular disease (CMD), its management in clinical practice, and the need for a CMD-specific guideline.
- The majority of cardiologists had considered the diagnosis of CMD; however, a much lower proportion viewed CMD as a separate disease entity.
- Although the opinion of male and female cardiologists differed regarding some aspects of CMD, this did not lead to differences in the management of patients with CMD or their interest in and/or their opinion concerning the need for a guideline.
- The majority of the responders would welcome a guideline on the diagnosis and management of CMD for Dutch cardiologists.

## Background

Coronary microvascular disease (CMD) is defined as the presence of signs and symptoms of ischaemia, in the absence of epicardial obstruction, with evidence of coronary microvascular dysfunction. It is a common condition in clinical practice, which affects both men and women [1–3]. Although CMD was previously thought to have a benign prognosis, recent studies have shown increased mortality among patients with CMD compared to patients without CMD. Furthermore, symptoms are often sustained and severe, giving rise to a diminished quality of life [4, 5]. Despite the reports regarding the large prevalence of CMD in clinical practice [3], the disease is often underdiagnosed, since the focus in ischaemic heart disease is still on coronary artery stenosis. As a consequence, CMD patients often undergo repeated (invasive) diagnostic tests and hospital admissions with associated high health care costs [6]. The European Society for Cardiology and the American Heart Association/American College of Cardiology acknowledge CMD as a separate disease entity and included recommendations for CMD in their latest editions of guidelines on stable coronary artery disease [7, 8]. However, the existing guidelines offer limited guidance on how to diagnose and treat these patients in clinical practice and mainly focus on symptom management.

So far, data concerning the Dutch cardiologists’ view with regard to CMD is lacking. Moreover, it is not clear whether developing a guideline on CMD would be welcomed by Dutch cardiologists. Therefore, our aim was to assess the opinion of Dutch cardiologists on CMD, its management in clinical practice in the Netherlands, as well as the need for a CMD guideline.

## Methods

We conducted a cross-sectional self-administered questionnaire survey among cardiologists in the Netherlands. A formal online questionnaire was developed using expert consensus. The questionnaire contained 26 items, consisting of 5 on CMD in clinical practice, 6 concerning the cardiologists’ view on and their knowledge of CMD, 5 regarding the need for a guideline on CMD, and 10 demographic items to characterise the study population (see the Electronic Supplementary Material for the questionnaire). The questionnaire was reviewed by an expert panel of the Gender Working Group of the Dutch Society of Cardiology (NVVC) and was validated in a representative group of individuals of the target population, among cardiologists across the Netherlands working in academic and/or non-academic hospitals. Next, the questionnaire was distributed to all members of the NVVC by e**-**mail together with the weekly newsletter. The questionnaire could be completed between 6 December 2018 and 15 January 2019. Participation was voluntary and anonymous.

Associations between survey responses and characteristics of the participants were evaluated by univariate analyses. χ^2^ tests were performed to compare dichotomous variables. The Mann-Whitney U test was performed for the comparison of non-normally distributed continuous variables. We further performed stratified analysis based on years of experience (dichotomised at the median) and also whether or not the cardiologists were currently involved in research. To test for non-response bias, we performed a sensitivity analysis in which we compared the two groups of early and late responders. Early responders were defined as those who had completed the questionnaire within 6 days after receiving the e‑mail. A two-sided *p*-value of <0.05 was regarded as statistically significant. Statistical analysis was performed with IBM SPSS Statistics software version 24.

## Results

Data are presented as frequencies and percentages for nominal variables, or medians and interquartile ranges for continuous variables.

### Demographics

The questionnaire was sent to 1905 members of the NVVC, of whom 1,044 were cardiologists. Of the 124 responders, 103 were cardiologists and 17 were cardiologists in training, cardiology residents not in training, or physician assistants. As this last group represented a minority, they were excluded. Therefore, the response rate was 10% among the cardiologists.

Among the responders, median (interquartile range) age and years of experience as a cardiologist were 49 ± 15 and 12 ± 12 years, respectively, and 70% were male. The majority of the responders had completed training in a sub-speciality (78%), including interventional cardiology (26%), non-invasive imaging (25%) or any other sub-speciality (23%), while 4% of the responders declared themselves to be specialised in ‘female cardiology’. A large proportion (65%) of the responders were currently involved in research, and 59% had a PhD degree. More than two-thirds (71%) of the responders were working in a non-academic hospital, 22% were working in an academic hospital, while 2% were working in both academic and non-academic hospitals, and 5% in a private clinic. The median (interquartile range) for the number of new patients per month was 200 (170), and the number of new patients with angina per month amounted to 30 (38) (Tab. 1).

**Table 1:** Demographics of responding cardiologists (*n* = 103)

	Cardiologists	Response rate
Age, years	49 (15)	99%
Sex, male	70%	100%^a^
Experience as a cardiologist, years	12 (12)	93%
Practice setting		100%^a^
– Academic hospital	22%	
– Non-academic hospital	71%	
– Both in academic and non-academic hospital	2%	
– Private clinic	5%	
Sub-specialty	78%	100%
– Interventional cardiology	26%	
– Non-invasive imaging	25%	
– Women’s heart health	4%	
– All others	23%	
PhD degree	59%	100%^a^
Currently involved in research	65%	100%^a^
Average number of patients per month	200 (170)	95%
Average number of new patients with angina per month	30 (38)	88%

^a^Response was obligatory

Data represent frequencies and proportions for categorical data and median (interquartile range) for continuous variables

### Opinion of cardiologists on diagnosis, prognosis and CMD as a disease entity

The median (interquartile range) self-rated level of knowledge among cardiologists was 7.0 (2.0) on a scale of 0 to 10. Of the responders, 84% rated their knowledge as sufficient (>5.5). Overall, 58% of the cardiologists viewed CMD as a separate disease entity. Although 61% agreed that evidence-based diagnostic modalities do not exist for the diagnosis of CMD, only 17% of responders agreed that treatment options do not exist. Moreover, 56% of cardiologists agreed that patients with CMD have a higher risk for cardiovascular disease and mortality (Tab. 2).

**Table 2:** Cardiologists’ view regarding coronary microvascular disease (*CMD*) as a disease entity and its diagnosis and prognosis (*n* = 103)

	Cardiologists
Self-rated knowledge	7.0 (2.0)^a^
*CMD is a disease entity*	
– Agree	58%
– Disagree	14%
– Do not know	28%
*Evidence-based diagnostic modalities to diagnose CMD do not exist*	
– Agree	61%
– Disagree	31%
– Do not know	8%
*Treatment options for patients with CMD do not exist*	
– Agree	17%
– Disagree	70%
– Do not know	15%
*Patients with CMD have a higher risk for cardiovascular disease and mortality*	
– Agree	56%
– Disagree	18%
– Do not know	26%

^a^On a scale of 0–10: 1 indicates very low, 10 indicates high

Data represent proportions for categorical variables and median (interquartile range) for continuous variables

The self-rated knowledge level did not differ significantly between male and female cardiologists. However, the opinion of male and female cardiologists differed considerably, as 51% of male cardiologists versus 74% of female cardiologists viewed CMD as a separate disease entity (*p* = 0.05). Also, 21% of male versus 7% of female cardiologists stated that treatment options do not exist (*p* = 0.03), and 44% versus 84% supported the statement that CMD leads to a higher cardiovascular morbidity and mortality (*p* = 0.001).

Responses to various questions differed significantly among cardiologists who viewed CMD as a disease entity compared to cardiologists who did not: 52% versus 79% stated that evidence-based diagnostic modalities do not exist (*p* < 0.0001), 8% versus 50% believed treatment options do not exist (*p* < 0.0001), and 77% versus 29% stated that CMD leads to a higher cardiovascular morbidity and mortality (*p* < 0.0001).

### Clinical practice

**Fig. 1:**
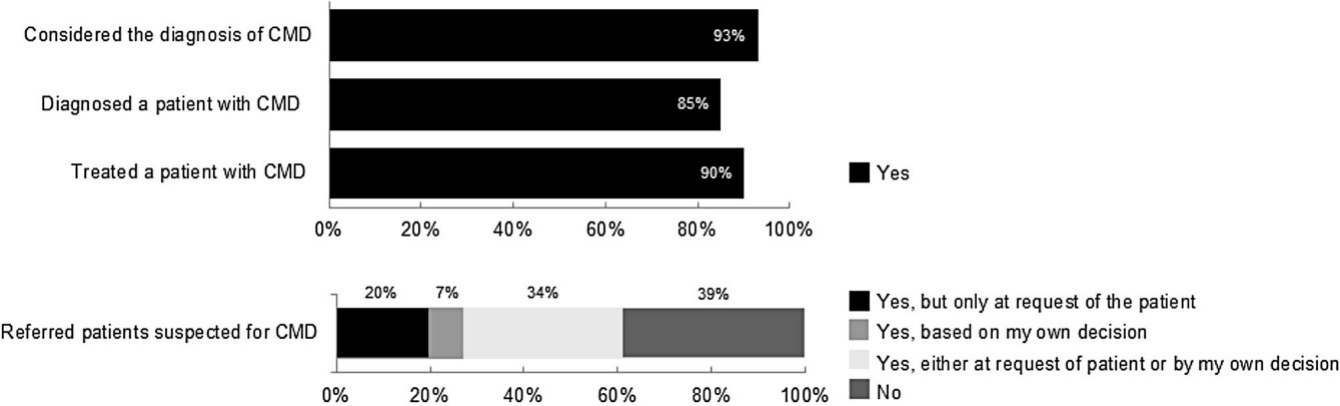
Experience of cardiologists regarding coronary microvascular disease (*CMD*)

Among the responders, the majority (93%) had considered the diagnosis of CMD in their practice (Fig. 1). Moreover, 85% and 90% of the cardiologists, respectively, had ever diagnosed or treated a patient with CMD in their clinic. Cardiologists who viewed CMD as a separate disease entity had more often considered the diagnosis, had ever diagnosed or treated CMD compared to cardiologists who did not: 98% versus 57% (*p* < 0.0001), 95% versus 43% (*p* < 0.0001) and 97% versus 57% (*p* < 0.001), respectively. Responses did not differ between cardiologists working in an academic versus a non-academic hospital.

Overall, 61% of the responders stated that they refer patients to third-line care or specialised clinics. One third of the responders referred patients based on both the request of patients and their own decision. Only 7% made a referral based on only their own decision, and 20% referred only at the request of the patients.

Among the responders that did not consider the diagnosis of CMD in clinical practice, a considerable proportion (43%) still stated that they had ever diagnosed or treated a patient with CMD in their own clinic or that they had referred patients.

Although more female than male cardiologists had ever diagnosed a patient with CMD (97% vs 80%, *p* = 0.02), responses regarding consideration of the diagnosis and patient referral for CMD did not significantly differ between the two genders.

### Treatment of CMD

**Fig. 2:**
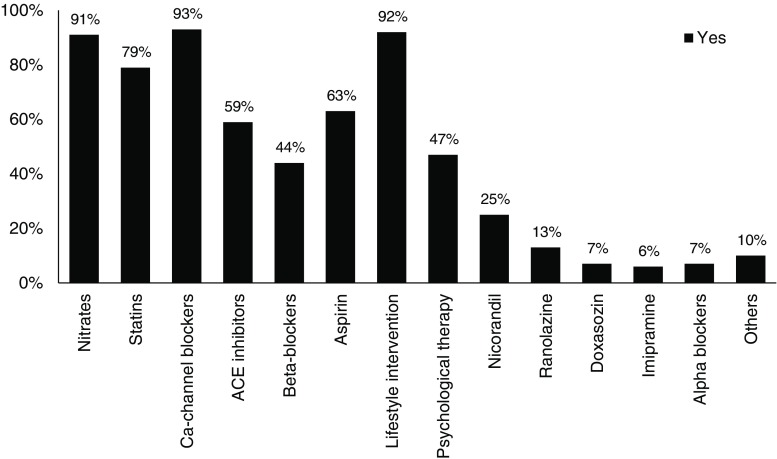
Treatment options used by Dutch cardiologists for coronary microvascular disease (*CMD*)

The most frequent used treatment options were calcium channel blockers, nitrates, statins, and lifestyle intervention, prescribed by 93%, 91%, 79%, and 92% of the responders, respectively (Fig. 2).

### Sex differences in CMD

**Fig. 3:**
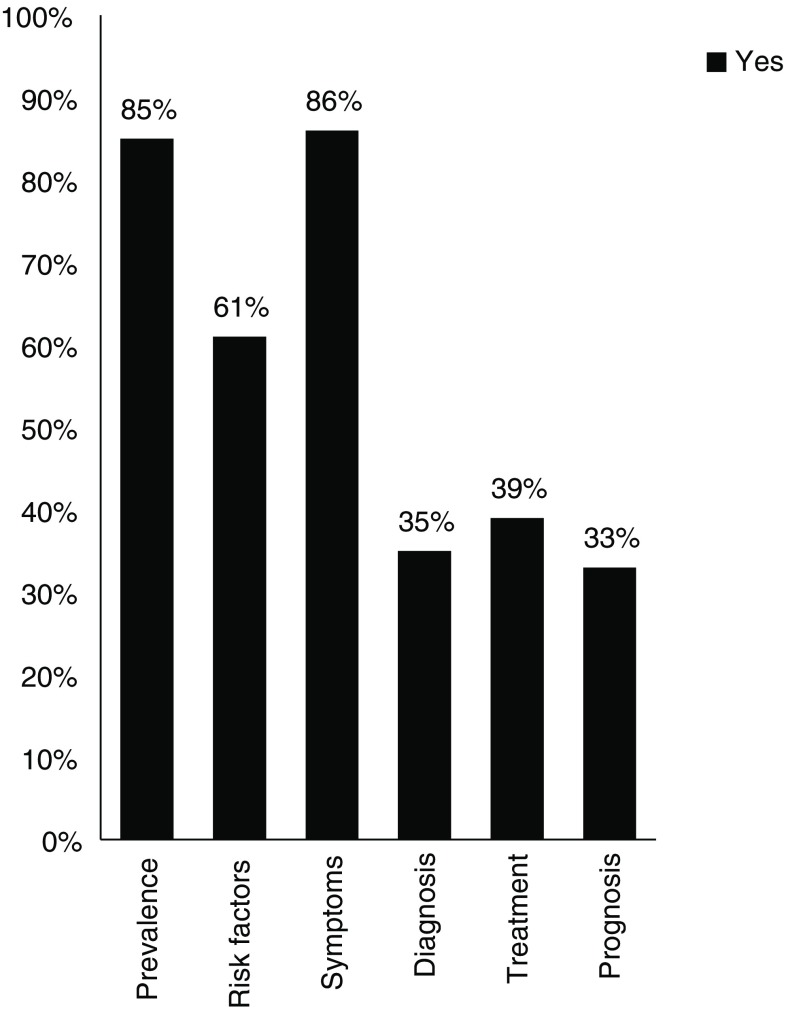
Cardiologists’ view regarding sex differences in coronary microvascular disease (*CMD*)

A large proportion of the responders (86%) indicated that sex differences exist for CMD: 86%, 85%, and 61% of responders stated that sex differences exist for prevalence, symptoms and risk factors in patients with CMD, respectively. However, a much lower proportion believed that sex differences are applicable to diagnosis, treatment, and prognosis (35%, 39%, and 33%, respectively). (Fig. 3).

### Guideline on CMD

The majority of responding cardiologists (82%) indicated that a guideline for CMD is needed, and an even larger proportion (91%) wanted to receive the guideline once developed. Compared to cardiologists practicing in academic hospitals, a larger proportion of cardiologists practicing in non-academic hospitals stated that there is a need for a CMD guideline (64% vs 87%, respectively; *p* = 0.01). In addition, 93% of cardiologists who viewed CMD as a separate disease entity, but only 50% of those who did not, responded that a CMD guideline was needed (*p* < 0.001). No differences were observed between male and female responders.

When responders were asked which topics should be included in the guideline, between 82% and 85% specified prevalence, prognosis, risk factors, and sex differences, while 95% indicated diagnosis and treatment.

## Discussion

We investigated the view of Dutch cardiologists on CMD, its management in clinical practice, and the need for a specific guideline for CMD. The results of the questionnaire underscore that the majority of the responding Dutch cardiologists would welcome a specific guideline for CMD.

### Interpretation of findings

The results from this survey should be interpreted in the context of several potential limitations. Firstly, the response rate was limited (10%). However, using the available demographic information of the total group of cardiologists that are members of the NVVC, the demographics of responders in the current study were comparable to those of the total group (70% vs 74% were male, 59% vs 56% had a PhD degree, and median age was 49 vs 45 years, respectively). Therefore, although the response rate was limited, our population of interest may still be representative of the total group of cardiologists. Secondly, although the target population was well represented, non-response might be related to the topic under study, and the responders may reflect those with a greater degree of interest and knowledge pertaining to CMD, thus leading to overestimation of the results. This might explain the rather high percentages for various questions observed in this study. However, a sensitivity analysis comparing early and late responders, assuming late responders behave similar to non-responders, yielded similar results. Another explanation might be that recent media attention in the Netherlands has been effective in raising awareness for CMD among cardiologists. Nevertheless, as the survey was dependent upon voluntary participation, and thus vulnerable for non-response bias, the true percentages remain unknown. Consequently, the generalisability of our results might be constrained by the characteristics of our sample population. Thirdly, as in most surveys, the present study relied on self-report and did not validate self-reports against objective measures.

### Strengths

We developed a questionnaire including a broad range of topics and covering different aspects of CMD which was reviewed by an expert panel. Moreover, the questionnaire was distributed by the NVVC. As almost all cardiologists in the Netherlands are members of the NVVC, this ensured widespread distribution across the Netherlands.

### Cardiologists’ view on CMD

Although the majority of the responders considered the diagnosis of CMD, a much lower proportion viewed CMD as a separate disease entity. In line with this, the latter group responded less positively to the question regarding the need for a specific CMD guideline. Thus, despite accumulating scientific evidence regarding CMD being a distinctive type of ischaemic heart disease, 58% of the responding Dutch cardiologists do not agree on this.

### Diagnosis and management of CMD in clinical practice

An important and encouraging observation from this survey is that the majority of physicians considered the diagnosis of CMD in their practice. Also, a large proportion of cardiologists preferred to manage patients with CMD in their own clinic by themselves. If our survey reflects the real clinical practice in the Netherlands, our results indicate that the management of CMD patients is not restricted to specialised centres. This emphasises the need for a CMD guideline for the clinical practice of cardiologists. Our study also showed that although the opinion of male and female cardiologists differed in some aspects, this did not lead to differences in the management of CMD in clinical practice or agreement on the need for a guideline.

### Implications of findings

Several aspects of our results highlighted the need for a CMD guideline. Firstly, a large percentage of cardiologists practicing in non-academic hospitals preferred to manage patients suspected of having CMD by themselves, rather than referring them to specialised centres. Secondly, our study highlighted differences in the management of CMD in clinical practice. Thirdly, although the self-rated knowledge was more than sufficient among the majority of the responders and despite the differences in opinion regarding CMD as a disease entity, the majority of responders agreed on the need for a guideline on CMD.

## Conclusion

Overall, 58% of the responders recognise CMD as a separate disease entity. The majority of responding Dutch cardiologists would welcome a guideline on the diagnosis and management of CMD in clinical practice.

## Caption Electronic Supplementary Material

Supplemental Material 1. Questionnaire, English Version
