# Supplementary material for: Questionnaire survey on cardiologists’ view and management of coronary microvascular disease in clinical practice
Source: Neth Heart J. 2019 Apr 12;27(5):252–62. doi: 10.1007/s12471-019-1274-x (PMC6470226; doi:10.1007/s12471-019-1274-x)
Supplement: Supplementary file 1 — Supplemental Material 1. Questionnaire, English Version [file 12471_2019_1274_MOESM1_ESM.docx]

**Supplemental Material 1.** Questionnaire, English Version

On a scale of 1 to 10; how would you rate your knowledge on coronary microvascular disease (CMD)?
 0  1  2  3  4  5 6  7 8  9 10

Do you consider the diagnosis CMD for patients with recurrent or persistent ischemic-like symptoms and no-obstruction in the epicardial coronary arteries?
 Yes
 No

Have you ever diagnosed a patients with CMD in your practice?
 Yes
 No

Have you ever treated a patients with CMD in your practice?
 No
 Yes

Which of the following treatment options do you use for the treatment of patients with CMD?
Nitrates
Statins
Calcium channel blockers
ACE inhibitors
Beta-blockers
Alfa blockers
Aspirin
Lifestyle intervention (diet and exercise)
Psychological therapy
Nicorandil
Ranolazine (if possible)
Doxasozin
Imipramine
Others

Have you ever referred patients suspected of CMD to tertiary care or specialized centers for CMD?
 Yes, but only at request of the patient
 Yes, based on my own decision
 Yes, both at request of the patient and/or by my own decision
 No **Give your opinion for the following statements:**
Coronary microvascular disease is a separate disease-entity
 Agree
 Disagree
 I don’t know

Evidence-based diagnostic modalities to diagnose CMD do not exist.
 Agree
 Disagree
 I don’t know

Evidence-based treatment options do not exist for patients with CMD.
 Agree
 Disagree
 I don’t know

Coronary microvascular disease leads to a higher risk for cardiovascular disease and mortality.
 Agree
 Disagree
 I don’t know

Sex-differences exist for coronary microvascular disease. For which of the following domains is this correct? (selecting multiple options is possible)
 Prevalence  Prognosis
 Diagnostics  Symptoms
 Treatment  Risk factors

There is a need for a specific guideline on CMD for cardiologist.
 Agree
 Disagree
 Others:                     

Explain your answer:
                                                                                

Which of the following topics should be addressed in this guideline? (selecting multiple options is possible)
 Prevalence  Prognosis  Sex differences
 Diagnostics  Symptoms
 Treatment  Risk factors

Are there any other topics or questions you would like to be included in this guideline?                                                                                 

If a guideline for CMD will be developed, I would like to receive it.
Yes
 No

**Demographics:**Your current profession:
 Cardiologist
 Cardiologist in training
 Resident cardiology not in training
 Physician assistant cardiology
 Others:                     

Sex:
 Men
 Women

Age:
                    

Where are you currently practicing?
 Academic hospital
 Non-academic hospital
 Both
 Private-clinic

For how long are you practicing as a cardiologist?
                     year

Do you have a sub-specialisation in cardiology?
 No
 Yes, interventional cardiology
 Yes, electrophysiology
 Yes, congenital cardiology
 Yes, intensive care
 Yes, non-invasive imaging
 Yes, others                                    

How many patients do you see on average per month?
                    
How many new patients with ischemia-like symptoms do you see on average per month?
                    

Do you have a PhD-degree?
 Yes
 No

Are you currently involved in research?
 Yes
 No
